# Evaluating the role of a trypsin inhibitor from soap nut *(Sapindus trifoliatus L. Var. Emarginatus)* seeds against larval gut proteases, its purification and characterization

**DOI:** 10.1186/s12858-015-0052-7

**Published:** 2015-10-22

**Authors:** V D Sirisha Gandreddi, Vijaya Rachel Kappala, Kunal Zaveri, Kiranmayi Patnala

**Affiliations:** Assistant professor, Department of Biochemistry/Bioinformatics, Institute of Science, GITAM University, Rushikonda, Visakhapatnam, 530045 Andhra Pradesh India; Department of Biotechnology, Institute of Science, GITAM University, Rushikonda, Visakhapatnam, 530045 Andhra Pradesh India

**Keywords:** K_i_, Monoheaded inhibitor, PAGE, SNTI, Specificity, Gut proteases, Protein-protein docking

## Abstract

**Background:**

The defensive capacities of plant protease Inhibitors (PI) rely on inhibition of proteases in insect guts or those secreted by microorganisms; and also prevent uncontrolled proteolysis and offer protection against proteolytic enzymes of pathogens.

**Methods:**

An array of chromatographic techniques were employed for purification, homogeneity was assessed by electrophoresis. Specificity, Ki value, nature of inhibition, complex formation was carried out by standard protocols. Action of SNTI on insect gut proteases was computationally evaluated by modeling the proteins by threading and docking studies by piper using Schrodinger tools.

**Results:**

We have isolated and purified Soap Nut Trypsin Inhibitor (SNTI) by acetone fractionation, ammonium sulphate precipitation, ion exchange and gel permeation chromatography. The purified inhibitor was homogeneous by both gel filtration and polyacrylamide gel electrophoresis (PAGE). SNTI exhibited a molecular weight of 29 kDa on SDS-PAGE, gel filtration and was negative to Periodic Acid Schiff’s stain. SNTI inhibited trypsin and pronase of serine class. SNTI demonstrated non-competitive inhibition with a Ki value of 0.75 ± 0.05×10-10 M. The monoheaded inhibitor formed a stable complex in 1:1 molar ratio. Action of SNTI was computationally evaluated on larval gut proteases from *Helicoverpa armigera* and *Spodoptera frugiperda*. SNTI and larval gut proteases were modeled and docked using Schrodinger software. Docking studies revealed strong hydrogen bond interactions between Lys10 and Pro71, Lys299 and Met80 and Van Der Waals interactions between Leu11 and Cys76amino acid residues of SNTI and protease from *H. Armigera*. Strong hydrogen bonds were observed between SNTI and protease of *S. frugiperda* at positions Thr79 and Arg80, Asp90 and Gly73, Asp2 and Gly160 respectively.

**Conclusion:**

We conclude that SNTI potentially inhibits larval gut proteases of insects and the kinetics exhibited by the protease inhibitor further substantiates its efficacy against serine proteases.

## Background

Plants commonly exhibit structural and biochemical defense mechanisms when challenged by pathogens and herbivores. With rising incidence of destructive activities of numerous pests like fungi, weeds and insects leading to radical decrease in yields, use of pesticides has become inevitable. An ecofriendly alternative to chemical pesticides is bio-pesticides, which encompasses a broad array of microbial pesticides, bio-chemicals derived from micro-organisms and other natural sources. One such compound is polypeptide that act upon the cell membrane of pathogens [1, 2]. They are capable of blocking protease mediated pathway by targeting the active sites of proteases by forming protease-inhibitor complex and results in breaking down of enzymatic activity [3] and hence these compounds are termed as protease inhibitors [PIs]. PIs are small proteins quite common in nature and these defense-related proteins are often present in seeds, and also induced in certain plant tissues by herbivory or wounding [4]. The defensive capacities of plant PIs rely on inhibition of proteases present in insect guts or secreted by microorganisms and reduce the availability of amino acids necessary for their growth and development. Protease inhibitors play an endogenous role in preventing uncontrolled proteolysis and/or in protecting against the foreign proteolytic enzymes of pests or pathogens [5, 6]. Observations of their wound-inducible expression [7, 8] have led to investigations focusing on their role in plant protection against insects [9–11].

Antifungal proteins contribute defense against pathogenic fungi. A variety of antifungal proteins were isolated from the seeds of leguminous plants, including French bean, cowpea, field bean, mung bean, peanut and red kidney bean. Nearly all leguminous antifungal proteins examined were able to inhibit HIV-1 reverse transcriptase, protease and integrase to some extent [12]. There is also a wide range of herbal ingredients which have been documented to have anti pityrosporum or anti dandruff activity [13]. Understanding the molecular mechanisms of natural plant products against dermatophytes could lead to the development of safe antifungal agents for controlling human skin diseases.

One of the most diverse species among living organisms on earth are insects [14]. One fifth of the total crop production is destroyed by insects [15]. Synthetic insecticides are most widely used to control the insects, but they in turn cause harmful effects on soil and plants [16]. In order to overcome such adverse effects of synthetic insecticides, there is a need for natural insecticides which are derived from plants or microorganisms. Allelochemicals are natural plant compounds which are invariably studied on plant-insect relationship [17]. Some protease inhibitors have shown anti insecticidal activity [18, 19]. Potential natural insecticidal compounds from different plants are identified which are referred to as trypsin inhibitors. The mid gut region of insect digestive system comprises of digestive proteases which catalyses the breakdown of proteins into small molecules [20]. Protease inhibitors are proficient in interfering with digestive enzymes of insect gut and hence are able in controlling them [21]. JSTI (Jack fruit Seed Trypsin Inhibitor) has effectively shown insecticidal activity against proteases of larval mid gut [18]. SSTI (*Sapindus saponaria* Trypsin Inhibitor) from *Sapindus saponaria* L., of the family *Sapindaceae* also exhibited substantial inhibitory activity against gut proteases of rice and flour moths, velvet bean caterpillar moth and sugar borer [19].

Ever since these inhibitors are identified, their role in medicinal and agricultural fronts are being extensively investigated. Accordingly, preliminary studies on protease inhibitors are carried out by screening different plant species (Table 1) and found *Sapindus trifoliatus* seed protease inhibitor to exhibit higher inhibitor activity among the group. Soap nut tree (*Sapindus trifoliatus L.*) belongs to the family of *Sapindaceae*, which is native primarily to tropical climate. It is an evergreen plant and most commonly found in South India whose fruits are rich in saponins [1, 22] and nuts are rich in Kaempferol, quercetin and β-sitosterol and is one of the well-known plant with rich medicinal values [23]. Soap nut is used for curing eczema, treating psoriasis and removing freckles. This herb is also used for removing lice from the scalp, since they have gentle insecticidal properties. The crushed seeds are widely used for making soaps and shampoos for their antibacterial, antifungal, stomachic and spermicidal activity. With a known potential for its medicinal properties the present study is carried out to purify and characterize this protease inhibitor from the seeds of soap nut for further applications.

**Table 1:** Protease inhibitor activities in different plant seeds

Name of the plant	% of Protease Inhibitory activity
*Annona squamosal*	46.60
*Achras sapota*	58.30
*Mimordica charantia*	57.14
*Moringa sp.*	11.10
*Trichosanthus sp*	Negligible
*Cucurbita maxima*	64.90
*Termenalia sp*	27.70
Vamu	66.60
Chironji	28.20
*Sapindus trifoliatus*	75.40
*Pomegranate*	40.00

The broad aim of this study is to isolate, purify and characterize protease inhibitor from Soap Nuts and computationally evaluate protease inhibitory action on larval mid gut proteases.

## Materials and methods

### Extraction and Purification of SNTIS

Sapindus trifoliatus trees bearing soap nuts were selected from Annavaram, East Godavari District, India. Ripe fruits are collected from selected trees and seeds are removed and preserved for extraction of protein. The endosperm was collected from the seeds after the removal of the hard coat and 25 g of the endosperm was homogenized with 200 ml of 0.1M sodium phosphate buffer, pH 7.6 and then made up to 250 ml with the same buffer. The extract was then centrifuged at 2500 rpm for 15 minutes at 4 ºC and the supernatant was used for further steps. The supernatant obtained was treated with 50 % ice cold acetone (1:5 v/v) and the resultant mixture was centrifuged at 2500 rpm for 15 minutes at 4 ºC. The precipitate was then re-suspended in 0.1M sodium phosphate buffer, pH 7.6.

The inhibition spectrum of SNTI was established by first assaying the protease or esterase activity of the enzyme on an appropriate substrate and then incubating a fixed amount of the enzyme with various amounts of the inhibitor and assaying the residual enzyme activity. The activities of trypsin and pronase or their inhibition were assayed by the method of Kakade et al., [24] using either BAPNA or casein as the substrate. The inhibitory activity towards chymotrypsin was determined using casein [25] or ATEE [26] as the substrate. The proteolytic activity of papain was assayed using casein as substrate by the method of Arnon [27]. The Esterolytic activity of subtilisin was assayedby using ATEE [28] as the substrate. Thermolysin was assayed according to the method of Matsubara [29]. The method of Saunders and Lang [30] was employed for assaying pancreatic α –amylase. Purification of the inhibitor is carried out by 50 % Ammonium Sulfate precipitation and dialyzed against PB (Phosphate Buffer). Further purification is achieved by ion exchange and gel permeation chromatography. Purity is assessed by native PAGE.50 % Ammonium Sulfate precipitation: The supernatant obtained after acetone fractionation was subjected to Ammonium Sulfate precipitation. Solid Ammonium Sulfate was added gradually with constant stirring at 4 ºC to obtain 50 % saturation. The mixture was allowed to stand overnight at 4 ºC.

Dialysis: The precipitate from 50 % Ammonium Sulfate precipitation was collected by centrifugation at 2500 rpm for 15 minutes at 4 ºC, then dissolved in 0.1 M sodium phosphate buffer pH 7.6 and dialyzed against the same buffer for 12 hours at 4oC. The dialysate obtained is subjected to column chromatography. The dialysate was further purified by column chromatography for separation of the inhibitor protein from the mixture of molecules based on charge and size using CM-cellulose and Sephadex G-100 columns.

Ion Exchange Chromatography: The dialyzed sample was loaded on a CM-Cellulose column (2×30cm) previously equilibrated with 0.1M sodium phosphate buffer pH 7.6. After washing with the equilibration buffer, stepwise elution was performed with increasing concentrations of 0.1M, 0.2M, 0.3M, 0.4M and 1.0 M NaCl and the respected fractions were collected. These fractions were monitored for protein by measuring their absorbance at 280 nm and fractions of each peak are pooled. Each pooled fraction samples were tested for the inhibitory activity against trypsin.

Gel Filtration Chromatography: The method of Andrews [31] was used to determine the molecular weight of the inhibitor by molecular sieve chromatography on Sephadex G – 100. Sephadex G-100 was swollen in 0.1M Phosphate buffer, pH 7.6 and packed in a column (2×30cm). The pooled fraction exhibiting inhibitory activity was loaded on Sephadex G- 100. The column was equilibrated and developed with the same buffer. The fractions were collected and the protein was monitored by measuring the absorbance at 280 nm”. The fractions from a single peak were pooled, dialyzed against phosphate buffer at 4 ºC and lyophilized.

High Performance Liquid Chromatography (HPLC): The column fraction with SNTI activity was then separated by reverse-phase HPLC, as described by Macedo et al., [19], on a C18 column(Shimadzu) that was previously equilibrated with water and >5 % acetonitrile. The SNTI active fraction was finally purified by rechromatography in a reverse phase HPLC with a flow rate of 1.0 ml/min for 35 min by isocratic elution. Proteins were monitored absorbance at 280 nm.

Polyacrylamide gel electrophoresis was carried out as described by Reisfield et al., [32] and followed by Gabriel [33]. PAGE was carried out under non-denaturing condition using 12 % slab gels. About 50 µg of protein was layered on the gel. After the electrophoretic run, proteins were fixed in 10 % TCA. Proteins were visualized using coomassie brilliant blue according to the method of Fairbanks [34].

### Characterization of SNTI

Molecular weight of the enzyme inhibitor: SDS Polyacrylamide gel electrophoresis was carried out using Phosphorylase-b (97.4 kDa), bovine serum albumin (66 kDa), ovalbumin (43 kDa), carbonic anhydrase (29 kDa), trypsin inhibitor (22.1 kDa) and lysozyme (14.3 kDa) as standard proteins for calibration.  The molecular weight of the inhibitor was determined using the calibration curve.

Effect of pH on the stability of the inhibitor: Solutions of the inhibitor (1mg ml-1) in 10 mM buffers of five pH values (pH 3.0, glycine-HCl; 5.0, sodium citrate; 7.0, sodium phosphate; 9.0, Tris-HCl; 12.0, glycine-NaOH) were kept at 50C for 24h. Aliquots of the inhibitor were diluted with 0.1M phosphate buffer, pH 7.6 and assayed for trypsin inhibitory activity (TIA).

Effect of temperature on the stability of the inhibitor: The inhibitor solutions (100µg ml-1) were separately incubated in a water bath at various temperatures for 10 min and then quickly cooled in ice and appropriate aliquots were assayed for trypsin inhibitory activity (TIA).

Kinetic measurements: The amidolytic activity of trypsin (50 µg) was determined with various concentrations of BAPNA (1.2to5.0µmol) in the absence of the inhibitor. The assays were then repeated in the presence of 5 and 15 µg of the inhibitor. The Ki values were calculated from Lineweaver-Burk plots.

Competition experiments: 50 µg trypsin was separately incubated with 5, 10, 15 and 20 µg of SNTI for 10 minutes at 37oC in 1ml phosphate buffer, pH7.6. Suitable aliquots of all the samples were taken and assayed for residual trypsin activity using BAPNA as a substrate. Studies on complex formation: The trypsin-SNTI complex was isolated by gel filtration on Sephadex G-100. To form the complex, 2 mg of SNTI was incubated with 5 mg of trypsin at 37oC for 15 minutes”.  Excess trypsin was used to make sure that all the inhibitor is complexed, such a mixture was applied onto a column of Sephadex G-100 at 5oC previously calibrated with SNTI and trypsin run separately and the absorbance was monitored at 280 nm. Trypsin and Trypsin inhibitory activities are monitored in the fractions collected.

Fourier Transform Infra-Red Spectroscopy (FTIR): The solid state FTIR spectra are recorded in the middle infrared (4000 cm-1to400 cm-1) on Perkin Elmer. The sample for FTIR analysis are prepared by grinding the dry blended powders of trypsin inhibitor with powdered KBr and then compressed to form discs.

Database and Sequence information: To demonstrate the inhibitory activity of SNTI on mid gut proteases, computational approach has been applied where in the proteases from larval guts of two insects viz., H. armigera and S. frugiperda were considered as these organisms commonly cause damage to agricultural fields. The sequence information for SNTI was taken from MALDI-TOF analysis done by Rachel et al., (2012) [35] and the gut proteases of H. armigera and S. frugiperda were retrieved from NCBI protein data base [36].

Homology modeling of SNTI and Threading based modeling of insect gut proteases: Primarily all the three sequences are subjected to PDB BLAST for template identification. The templates are identified based on homology and the homologous template was used for homology modeling of SNTI sequence. For the insect gut proteases no homologous templates were available, hence they are subjected for modeling using threading approach. In threading approach folds (secondary structure) of the protein are considered and templates are identified from fold library. Based on the folds of template identified the target protein is modeled. Prime module from Schrodinger suite (Schrodinger 2011) was used for modeling proteins by Homology and Threading approaches.

Structure Validation: The predicted structures are subjected for validation to ERRAT and PROCHECK servers. The validations by PROCHECK were done based on the stereo chemical quality, hydrogen bonding energy and torsion angles [37]. Based on the interaction of atoms with respect to amino acid residues ERRAT validates the predicted protein structure by separating correct and incorrect determined structures [38].

Binding Site prediction: The active site of all the three modeled proteins were predicted using SiteMap [39]. SiteMap determines primary binding site on a receptor by calculating the sites on protein surface by searching the grid points called site points. Then the contour site maps are generated, producing hydrophobic and hydrophilic maps.

Protein-Protein Docking: To understand role of SNTI in inhibiting proteases, modeled SNTI and gut proteases were subjected for protein-protein docking using Piper [40]. Prior to docking the proteins are subjected for protein preparation to optimize the molecule using PrepWizard. For protein-protein docking gut proteases were set as ligands and docked separately with receptor SNTI. Number of ligand rotation to probe were set for 10000 rotation and for each dock ten poses were retrieved. After docking, the best pose was selected and then these complex structures were again set for optimization by PrepWizard.  These prepared complexes are set for analysis of protein-protein interaction using protein interaction option in Bioluminate [41].

## Results

### Extraction and Purification

of SNTI The Soap Nut Trypsin Inhibitor was isolated and purified from soap nut seeds (Sapindus trifoliatus L.) according to the procedure adopted by Annapurna and Siva Prasad [42] and the results are shown in the Table- 2.

The extraction procedure was carried out maintaining physiological conditions and ice cold acetone was used to remove lipids. The endosperm was collected from the seeds after the removal of the hard seed coat and 25 g of the endosperm was homogenized with 200 ml of 0.1M sodium phosphate buffer, pH 7.6 and then made up to 250 ml with the same buffer. The extract was then centrifuged at 2500 rpm for 15 minutes at 4 ºC and the supernatant (230 ml) was used in further steps.

**Table 2:** Summary of purification of soap nut seed protease inhibitor

Preparation	Volume (ml)	Total protein (mg)	Total Trypsin inhibitory activity (Units) TIU0 × 10^3^	Specific activity TIA × 10^2^ (Units/mg) protein	Yield %	Fold purification
Crude extract	250	4030	63.10	0.156	100	1.00
Acetone fractionated extract	225	2644	54.81	0.207	86.60	1.33
Heat treated extract	200	2334	53.10	0.227	84.15	1.44
50 % Ammonium sulphate	30	172.5	23.78	1.370	37.69	8.78′
CM – Cellulose	27	112	14.74	1.440	23.36	9.23
Sephadex G-100	12	52	13.2	2.54	20.92	16.28

Yield and fold purification were calculated on the basis of TIU and TIA respectively

*TIU* trypsin inhibitory Units, *TIA* trypsin inhibitory activity

The supernatant (230 ml) was treated with 50 % ice cold acetone (1:5 V) and the resultant mixture was centrifuged at 2500 rpm for 15 minutes at 4 ºC to remove lipids. The resultant defatted solution was subjected to ammonium sulphate precipitation.

To the supernatant (200 ml) from acetone fractionation, solid ammonium sulphate (62.6 g) was added gradually with constant stirring at 4 ºC to obtain 50 % saturation. The mixture was allowed to stand overnight at 4 ºC. The precipitate was collected by centrifugation at 2500 rpm for 15 minutes at 4 ºC, then dissolved in 30 ml of 0.1 M sodium phosphate buffer pH 7.6 and dialyzed against the same buffer.

Proteins have numerous functional groups that can have both positive and negative charges. Ion exchange chromatography separates proteins with regards to their net charge. If a protein has a net positive charge at pH 7, then it will bind to a column of negatively charged beads, whereas a negatively charged protein would not. By changing the pH so that the net charge on the protein is negative, it too will be eluted.

The dialyzed sample (172 mg) was loaded on a CM-Cellulose column (2×80cm) previously equilibrated with 0.1M sodium phosphate buffer pH 7.6. After washing with 250 ml of the equilibration buffer, the following stepwise elution was performed with 200 ml each of 0.1M, 0.2M, 0.3M, 0.4M and 1.0 M NaCl in 0.1 M phosphate buffer pH 7.6. Fractions of 5 ml were collected at a flow rate of 60 ml per hour. These fractions were assayed for protein by measuring their absorbance at 280 nm as well as the inhibitory activity against trypsin using BAPNA as the substrate. The elution profile of CM-Cellulose chromatography for the inhibitor is shown in Fig. - 1. The fractions containing trypsin inhibitory activity (fractions 42-48) were pooled, dialyzed against distilled water at 4 ºC and lyophilized. The protein yield from ion exchange chromatography was 112 mg.

The sample from ion exchange chromatography (110 mg) was dissolved in 0.1 M phosphate buffer pH 7.6 and was loaded on Sephadex G-100 column (1.8 × 30 cm) which was previously equilibrated with 0.1 M phosphate buffer, pH 7.6. The inhibitor was eluted with the same buffer. 2 ml fractions were collected at a flow rate of 12 ml per hour and the protein was monitored by measuring the absorbance at 280 nm. The trypsin inhibitory activity of the fractions was assayed using BAPNA as the substrate.

**Fig. 1:**
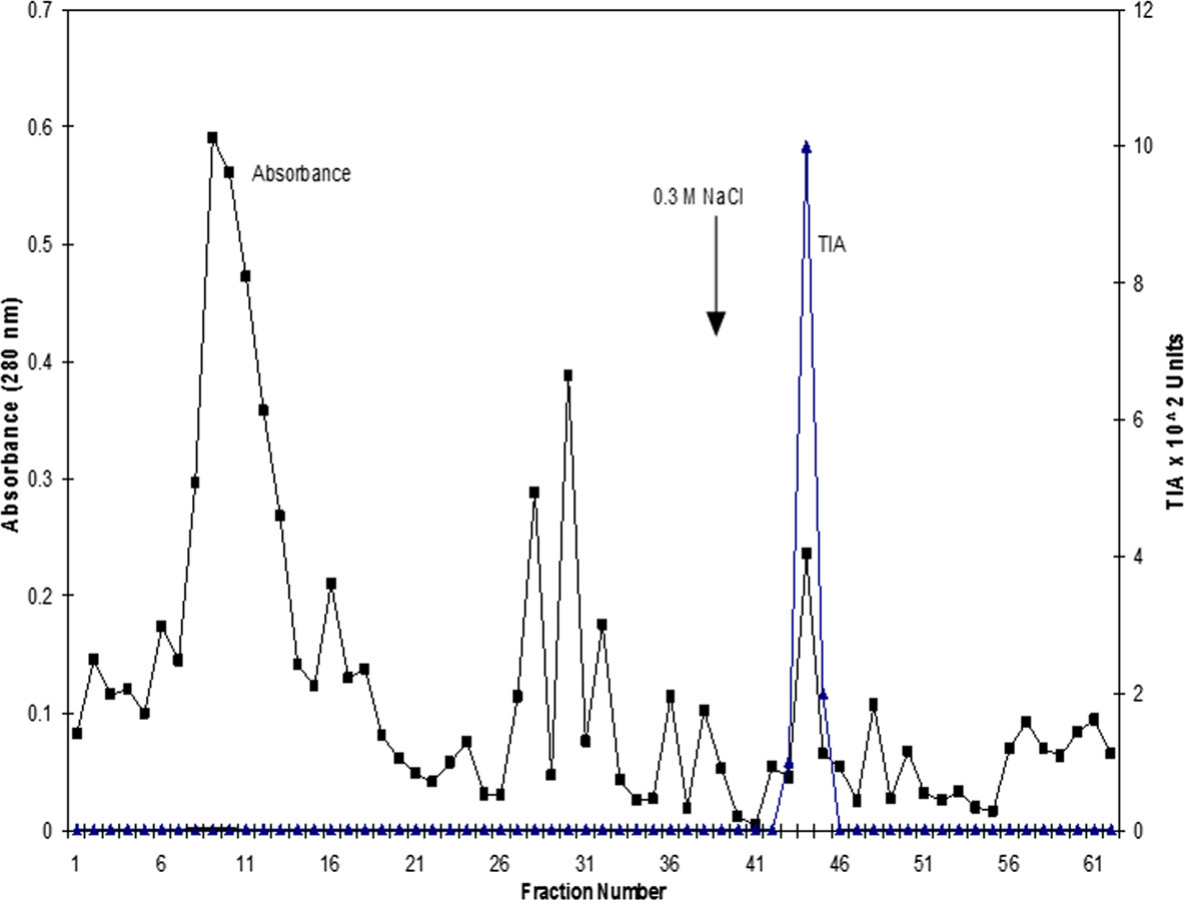
Ion exchange chromatography of SNTI on CM-Cellulose. One hundred seventy two milligram of the ammonium sulphate fractionated sample (0–50 %) was applied on to the column (2 × 80 cm) in 0.1 M sodium phosphate buffer (pH 5.8) and the adsorbed proteins were eluted with stepwise gradient in the buffer. Fractions of 5 ml were collected at a flow rate of 60 ml per hour. The protein was monitored by absorbance at 280 nm. * When the elution was done with a gradient of 0.1 to 1.0 M NaCl a single but broad peak was obtained (Results not shown. To obtain a sharp peak, the elution was performed using stepwise gradient

The elution profile of the gel permeation chromatography is shown in Fig. - 2. A single protein peak with corresponding trypsin inhibitory activity was observed. The fractions (8 - 12) containing the trypsin inhibitory activity were pooled, dialyzed against distilled water at 4 ºC and lyophilized. The yield of protein after gel permeation chromatography was 52 mg. This preparation was stored at 0 ºC. The preparation thus stored, showed full activity even after three months. By this procedure about 52 mg of the inhibitor was obtained and the final yield was about 20.9 %.

**Fig. 2:**
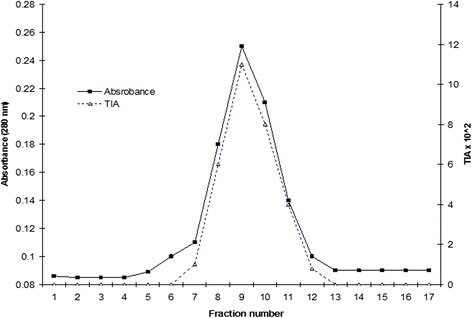
Gel filtration of SNTI on Sephadex G100. One hundred ten milligram of lyophilized preparation was applied to the Sephadex G-100 column 2 × 80 cm in 0.1 M phosphate buffer pH 7.6 and eluted with the same buffer. 2 ml fractions were collected at a flow rate of 12 ml per hour. The protein was monitored at 280 nm. Protease inhibitory activity was followed using BAPNA as the substrate

SNTI was analyzed using reverse phase HPLC to confirm its purity. HPLC analysis revealed a single peak (result not shown). The methodological procedure resulted in high purification with a 20.92 % yield.

A sharp band was obtained on 12 % slab gel at pH 8.3 signifying the homogeneity of the purified SNTI (Fig. - 3). SNTI did not respond to PAS (Periodic Acid Schiff’s) stain suggesting it to be a non-glycoprotein.

**Fig. 3:**
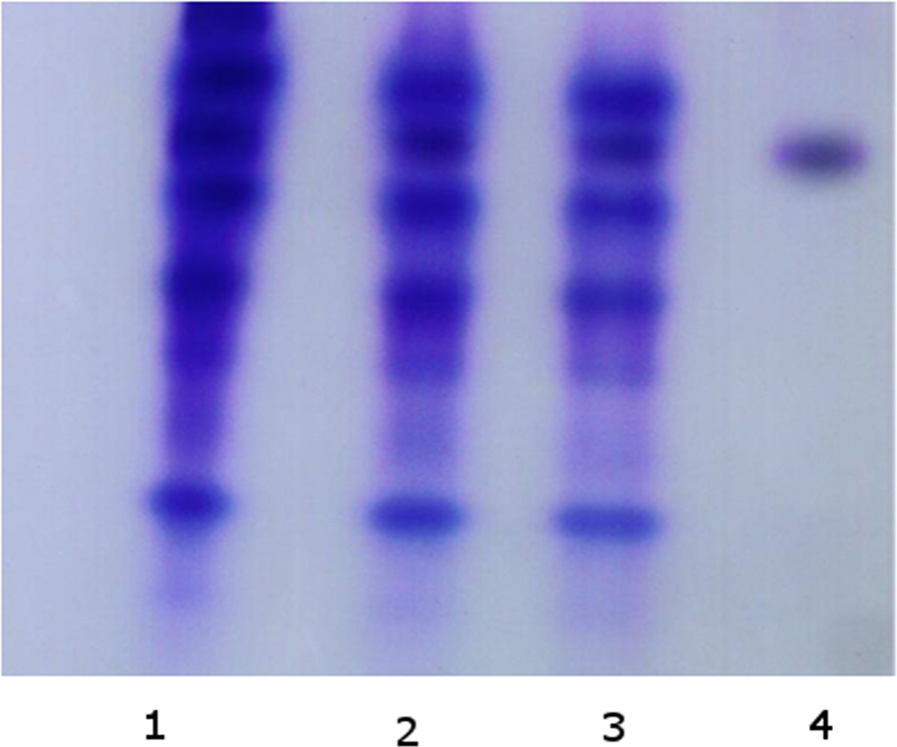
Polyacrylamide Gel Electrophoresis. 1 – Crude Extract. 2 – Acetone Fractionated. 3– Dialysate form Ammonium Sulphate precipitation. 4 – Sephadex Purified Sample

### Characterization of SNTI

Figure – 4a shows the protein band pattern of the inhibitor on 12 % SDS slab gels when stained with coomassie brilliant blue. Silver staining of SNTI showed a sharper band on SDS-PAGE. From the plot of distance migrated in cm versus log molecular weight for standard proteins (Fig. – 4b), the inhibitor showed a molecular weight of 29 kDa. When subjected to Gel filtration on Sephadex G-150, SNTI eluted out as a single protein with a corresponding activity peak (Fig. – 5a). The plot of elution volume versus log molecular weight of the calibrating proteins is shown in Fig. – 5b. The molecular weight of SNTI calculated from the plot was 28.5 kDa.

Enzyme inhibition studies were carried out to identify the specificity of the inhibitor towards the mechanistic classes of proteases. SNTI was tested for its inhibiting capacity against bovine trypsin using both BAPNA and casein as the substrates. The inhibition patterns of the amidolytic activity of bovine trypsin by SNTI was linear up to 80 % inhibition (Fig. - 6). On extrapolation, it was found that 12 µg of the inhibitor can totally inhibit amidase activity of 30 µg of trypsin.

The activity of the SNTI against chymotrypsin, elastase and pronase (Streptomyces griseus protease) subtilisin, papain, pepsin, thermolysin and α-amylase was tested. Except pronase, the rest of the enzymes were not affected by SNTI.

**Fig. 4:**
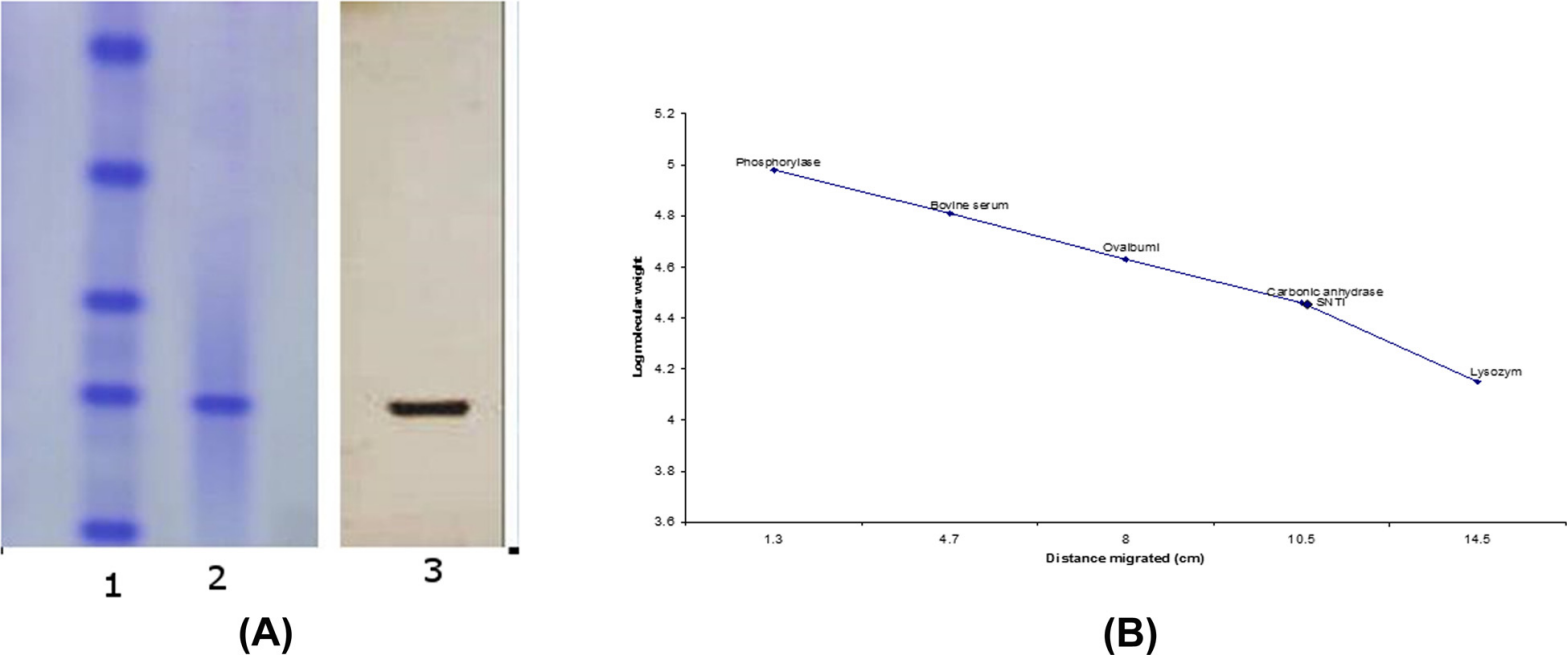
**a** SDS – PAGE. Direction of migration is from top (cathode) to bottom (anode). (1) Molecular weight markers: Phosphorylase b (97.4 kDa), Bovine serum albumin (66 kDa), Ovalbumin (43 kDa), Carbonic anhydrase (29 kDa), Lysozyme (14.3 kDa). (2). Purified SNTI (coomassie brilliant blue stained). (3) Purified SNTI (silver stained). *(1) to (3) were kept at 100 °C for 3 min with SDS and 2 mercaptoethanol. **b** Molecular weight determination of SNTI by SDS PAGE on 12 % slab gel. Plot of distance migrated against log molecular weight of standard proteins. BSA −66 K.Da Ovalbumin −43 K.Da Carbonicanhydrase −29 K. DaLysozyme −14.3 K.Da

**Fig. 5:**
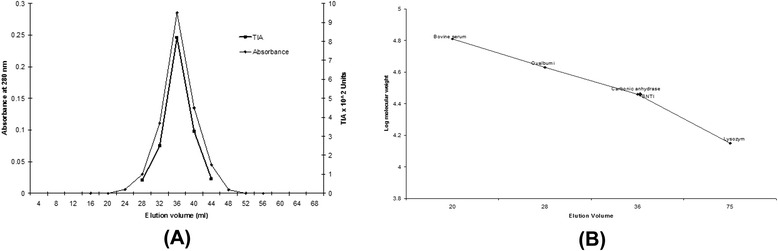
**a** Gel filtration of SNTI on Sephadex G-150. Elution profile of SNTI on a calibrated column of Sephadex G-150. 10 mg of purified SNTI was applied to the column in phosphate buffer pH 7.6 containing 20 mM NaCl and eluted with same buffer. Fractions of 4 ml each were collected at flow rate of 12 ml per hour. Protein was monitored at 280 nm. **b** Molecular weight determination of SNTI by gel filtration on Sephadex G-150. Plot of elution volume against log molecular weight of standard proteins. BSA −66 K.Da Ovalbumin −43 K.Da. Carbonicanhydrase −29 K.Da Lysozyme −14.3 K.Da

**Fig. 6:**
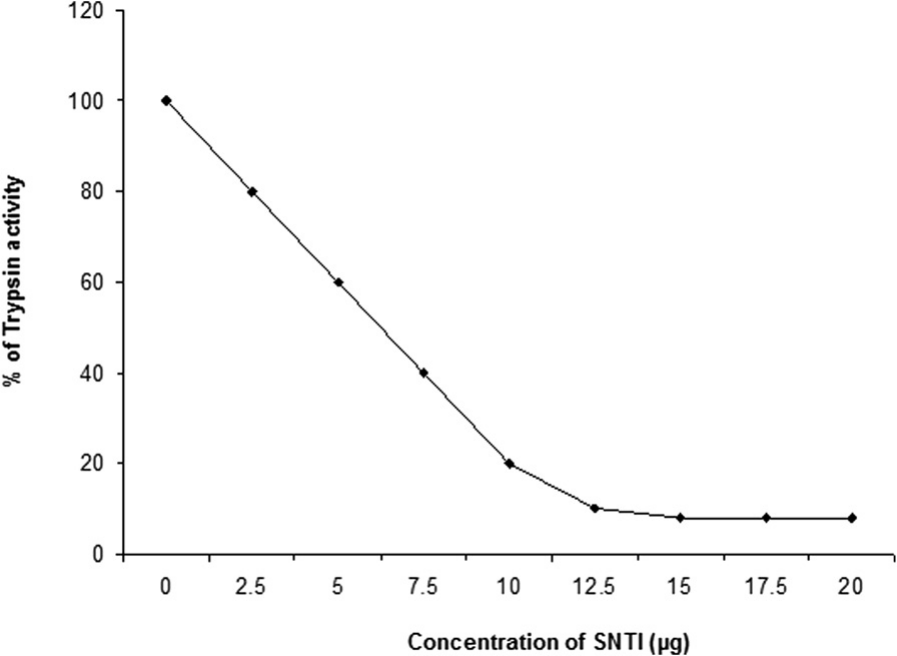
Activity of SNTI towards Bovine Trypsin. Thirty microgram of trypsin was incubated with varying amounts of SNTI for 10 min at 37 °C. The percentage residual enzyme activity was assayed using BAPNA as the substrate. The concentration of the inhibitor required to cause 50 % inhibition of the enzyme activity was determined from the graph

The serine proteases trypsin and pronase were inhibited by SNTI. Majority of plant protease inhibitors isolated so far have been found to be specific for serine proteases and there are some reports of these inhibitors inhibiting other classes of proteases. SNTI specifically inhibited serine proteases trypsin and pronase and it has no effect on thiol, acidic, metalloproteases and α-amylase.

Reverse zymography: Substrate containing SDS- PAGE enables visualization of trypsin inhibitor. The inhibitory activity produced by SNTI detected using trypsin and gelatin substrate in the gel is shown in Fig. - 7. SNTI showed a single inhibitory band specific to trypsin and subjected to electrophoresis.

Mode of inhibition of Trypsin: Trypsin activity in the presence and absence of SNTI was measured at different substrate concentrations. The double reciprocal plot of the kinetic data is shown in Fig. - 8. In the presence of inhibitor, there was a decrease in the Vmax and the curves met on the X –axis at a point equivalent to -1/km. The mode of inhibition of trypsin by SNTI was non-competitive. The Ki value of trypsin for SNTI calculated from Dixon plot was 0.75 + 0.05 × 10­-10 M.

Complex Studies: SNTI was treated with excess trypsin and the mixture was pre-incubated at 37oC for 15 minutes. This mixture when applied onto a column of Sephadex G-150 at 5oC gave rise to two distinct at 280nm peaks (Fig. - 9). Peak-I had an elution volume of 20 ml which is higher than free SNTI 35 ml. The binary complex of trypsin - SNTI did not show any trypsin activity or trypsin inhibitory activity.

The molecular weight calculated for trypsin – SNTI complex on Peak-I based on the calibration curve for standard proteins (Fig. – 4b) gave a value of 68.9 kDa. This would mean a mole/mole interaction of SNTI with trypsin. Peak-II was small and represented uncomplexed SNTI with corresponding trypsin inhibitory activity. The trypsin left over after the enzyme inhibitor complex formation, was eluted out as peak-III with a corresponding elution volume of 51 ml.

**Fig. 7:**
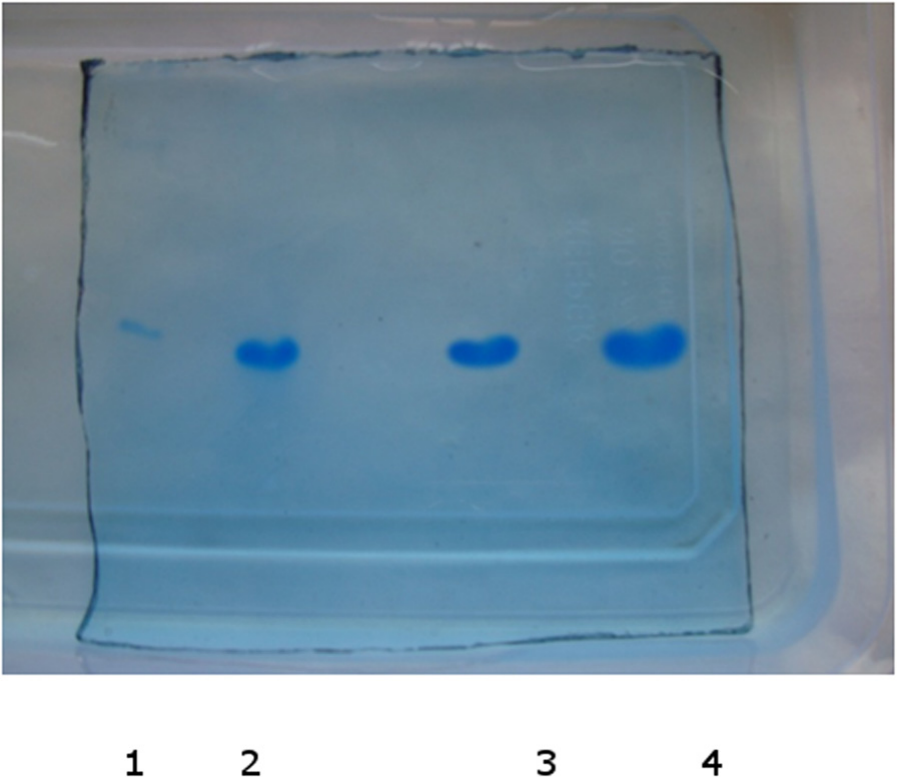
Reverse zymogram. 1. Crude fraction. 2. Ammonium Sulphate dialysate. 3. CM-Cellulose sample. 4. Sephadex purified sample

**Fig. 8:**
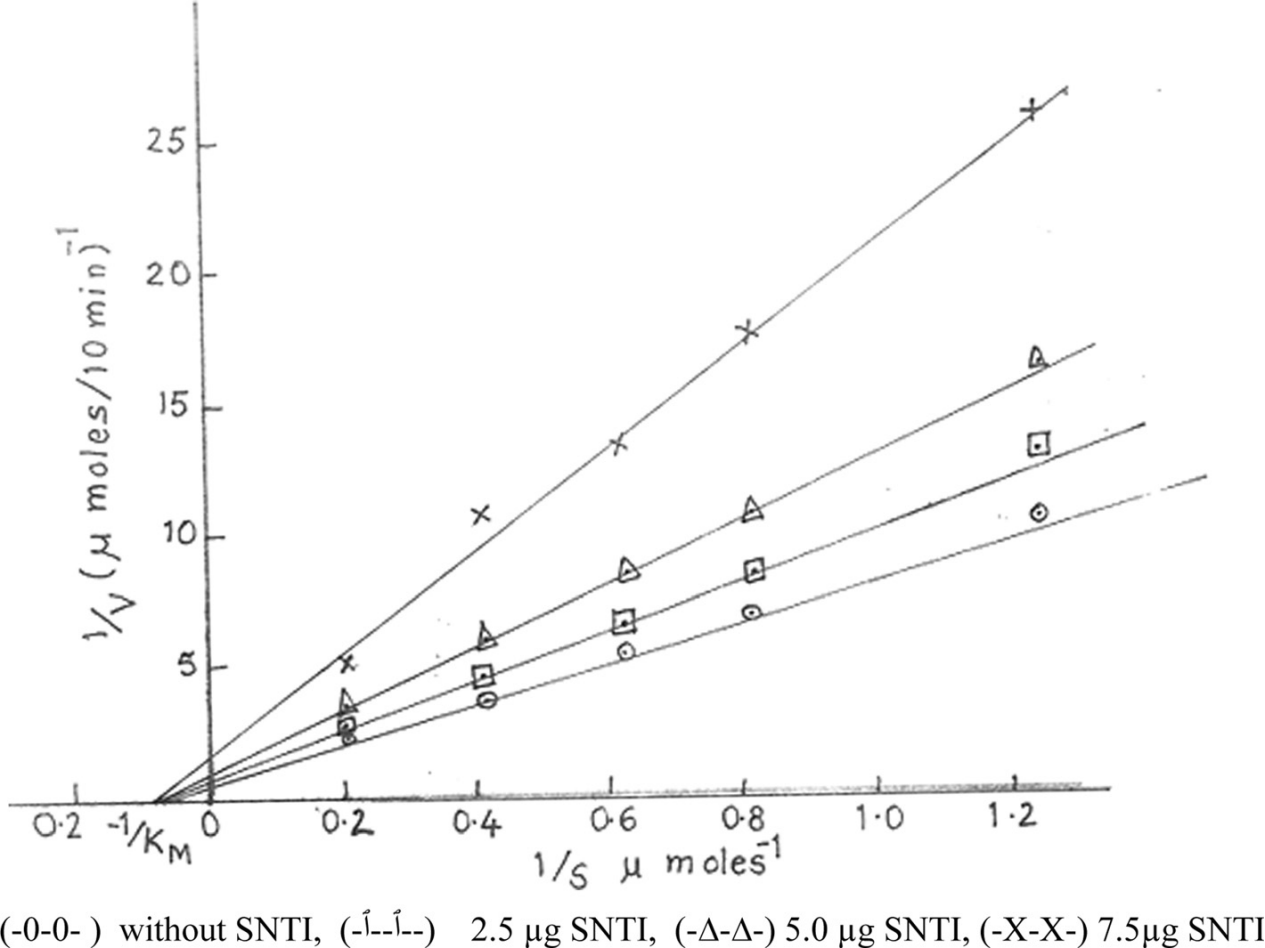
Mode of inhibition of trypsin by SNTI Line weaver –Burk plot. Inhibition of amidolytic activity of trypsin by SNTI was done by incubating 30 μg of trypsin and BAPNA solution (0.8 to 5.0 μ mole) with reaction system containing 2.5 to 7.5 μg of SNTI

**Fig. 9:**
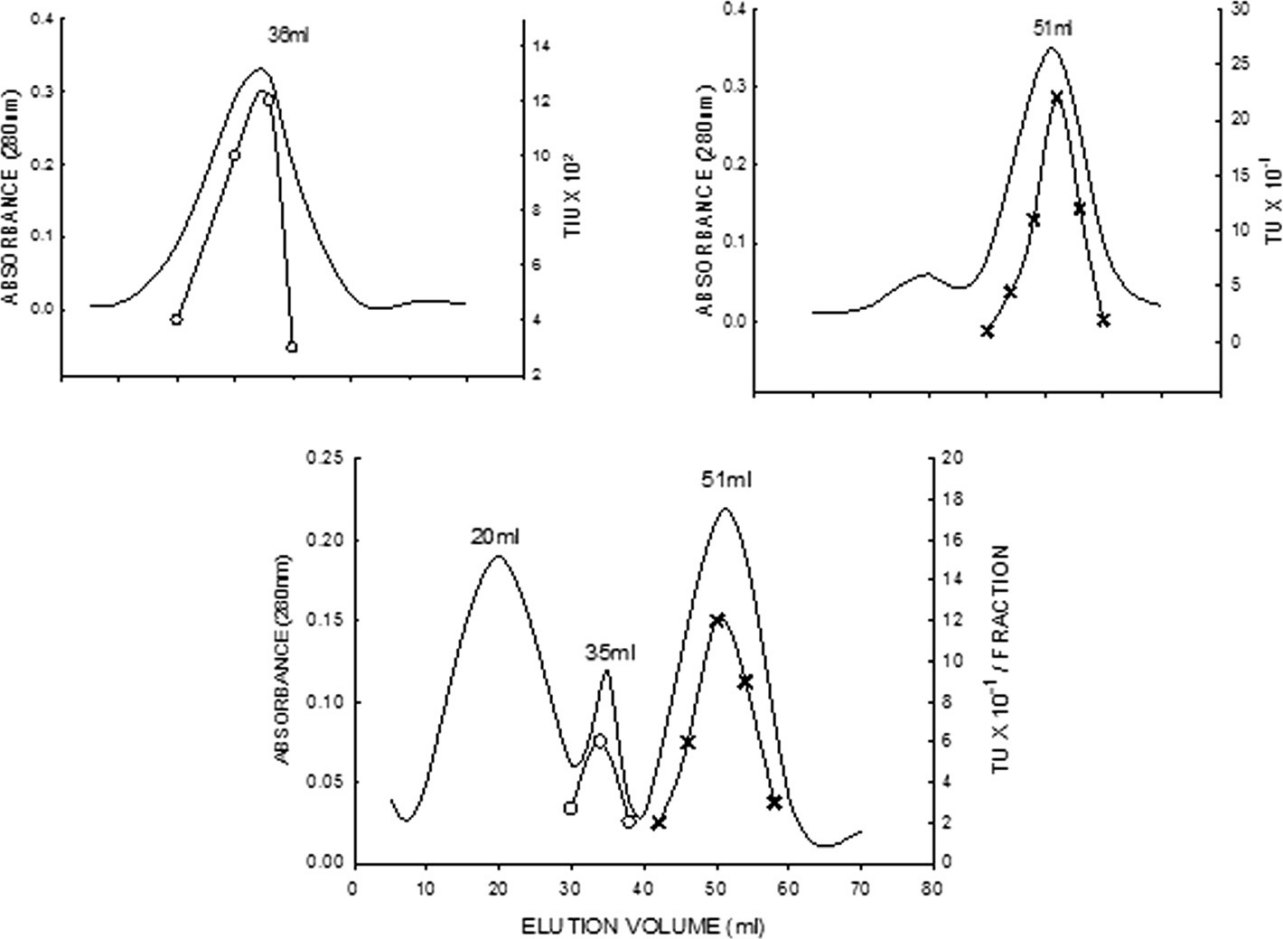
Elution patterns of SNTI, Trypsin and Trypsin-SNTI complex on Sephadex G-100 column. Elution patterns of trypsin and trypsin – SNTI complex on Sephadex G – 100. Protein was monitored at 280 nm

Fourier Transform Infra-Red Spectroscopy (FTIR): IR spectroscopic studies elucidates functional groups in a molecule. The IR peak at 3399 (broad) and 2939 cm-1can be assigned to OH of carboxylic group and asymmetric CH3 stretching. The over ton peak can be observed at 2074 cm-1. The peak at 1642 and 1423 cm-1 could be due to amide C=O (CONH2) and CH3 bending vibrations. The peaks observed at 995 and 925 could be attributed to OH bending vibrations. The presence of amide and carboxylic groups are confirmed by the above peaks (Fig. - 10).

Database and Sequence information: Protein sequences of gut proteases of H. armigera and S. frugiperda were retrieved from protein NCBI database bearing the accession number AHX25877.1 (Kazal-type serine protease from H. armigera) and ACR25157.1 (Trypsin protease from S. frugiperda).

**Fig. 10:**
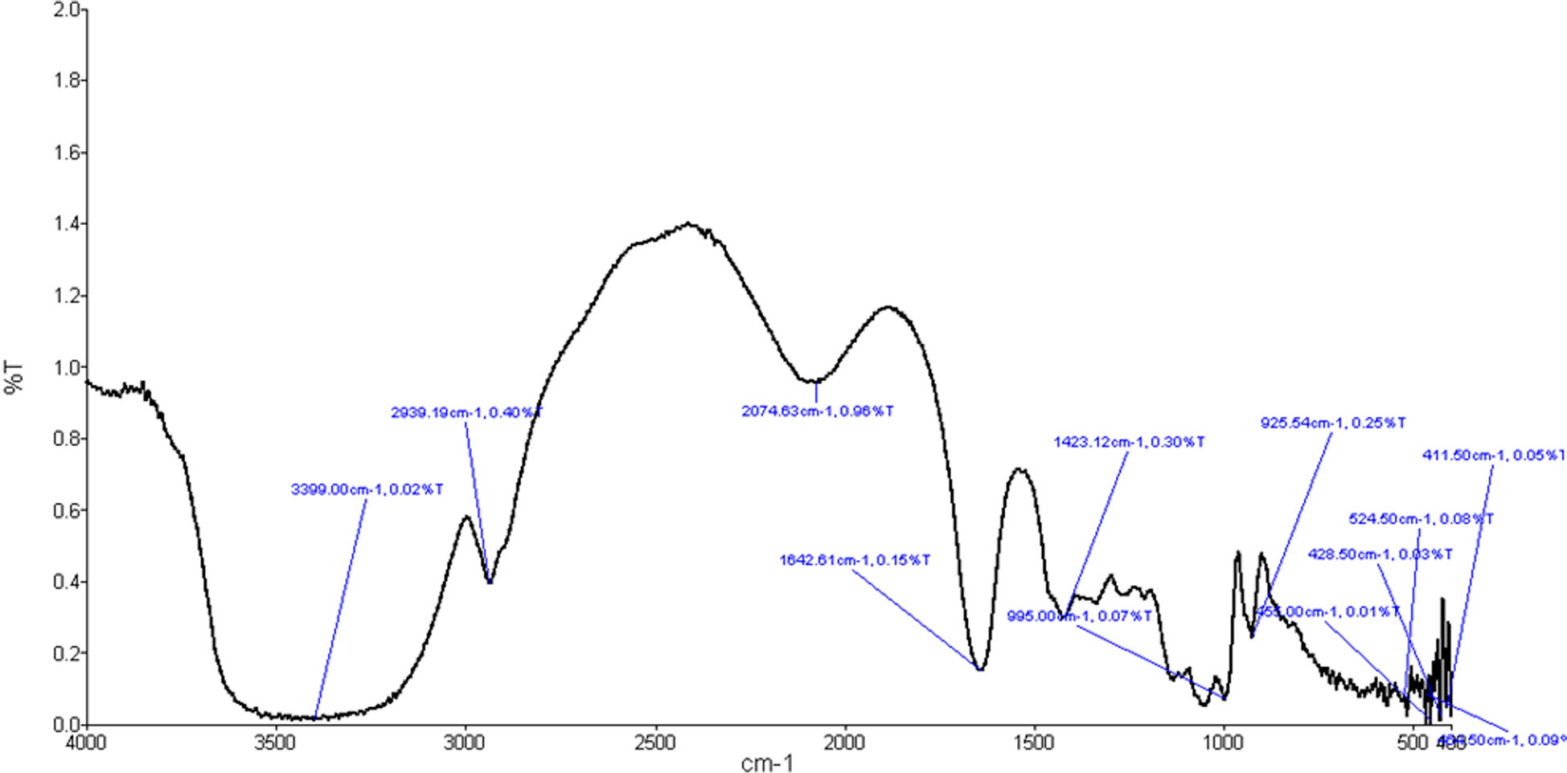
FTIR. FTIR peaks representing presence of amide and carboxylic groups

Homology modeling of SNTI and Threading based modeling of insect gut proteases:

The protein structures of all the three were modeled using Prime module from Schrodinger Suite. PDB BLAST provided a template 2C1X_A (UDP-Glucose Flavonoid 3-O Glycosyltransferase) with 42 % identities, 55 % positives and score of 192.6 for SNTI. Secondary structure of target SNTI sequence was identified using run SSP. After secondary structures for target are identified, template and target sequences are aligned and then the structure of SNTI is modelled based on the template 2C1X_A and the structure represents 11 helices and 8 beta sheets (Fig. - 11).

The target proteins Kazal type serine protease from H. armigera and trypsin protease from S. frugiperda are subjected to BLAST search for the identification of homologous template. Template structures with very low identity were retrieved so, instead of homology modeling threading or fold recognition approach was further used to model these proteases. In threading first the secondary structure of target protein sequences were predicted using run SSP option. Based on these secondary structures template is identified from the fold library and the best templates identified were crystal structure of insect derived kazal complex of serine protease (1TBQ) and crystal structure of a non-psychrophilic trypsin (1A0J) respectively.

**Fig. 11:**
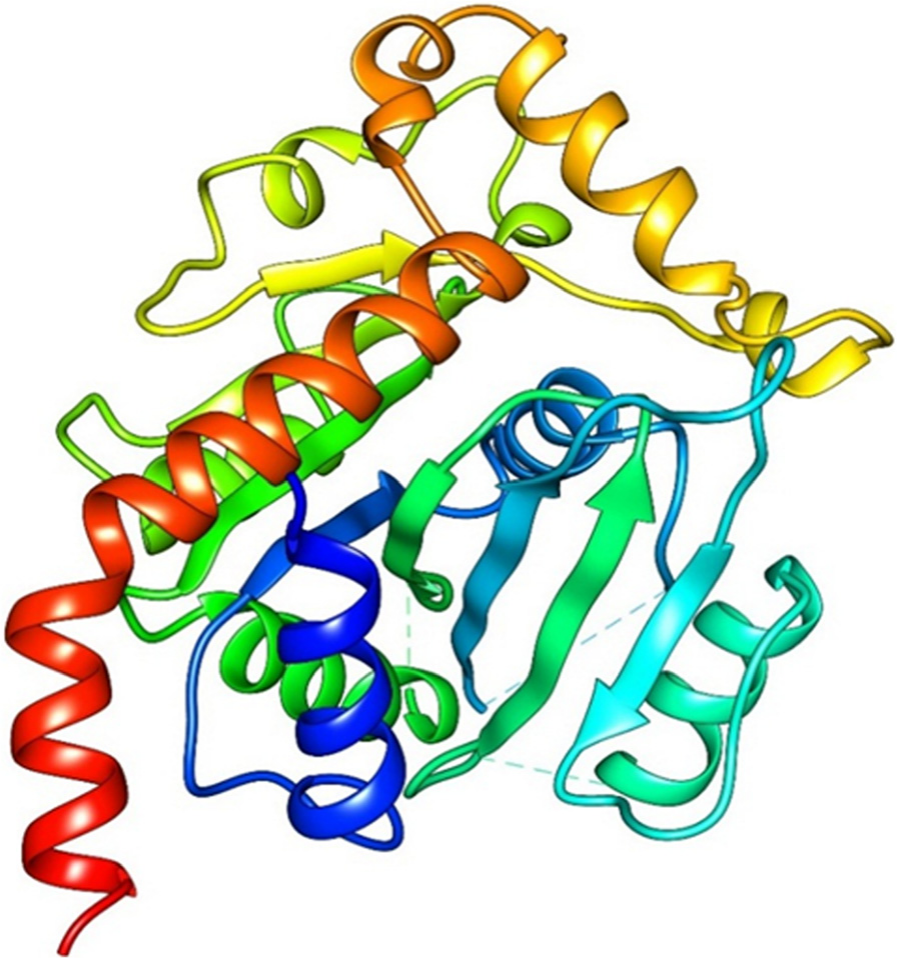
Modeled Structure of SNTI by Homology modeling. Homology modeled structure of SNTI representing 11 helices and eight sheets

These templates were further used for modeling Kazal type Serine and Trypsin proteases by homology modeling approach. Now, again the same first step is repeated but instead of finding the homologs, the template structure predicted by threading is used and the sequence of template and targets are aligned. Finally the model was built for Kazal type serine protease from H. armigera and was found to have 4 helices and 3 beta sheet (Fig. - 12). Similarly, Trypsin from S. frugiperda has 5 helices and 3 beta sheets (Fig. - 13). The 3D structure obtained is then validated using PROCHECK and ERRAT (Fig. - 14a, 14b). The protein structure that is modeled is satisfactory as evidenced by the validation tools. Ramachandran plot derived from PROCHECK analysis represents about 99.2 % of amino acids residues of SNTI are in favored region (Fig. - 14a) and ERRAT validates the overall structure quality to be 86.029 % (Figure - 14b). About 92.5 % of amino acids residues falling in favored regions for Kazal type Serine protease (Figure - 15a) and 97.5 % for trypsin protease (Fig.- 16a). ERRAT validates the overall structure quality of Kazal type Serine protease to be 86.96 % (Fig.- 15b) and 81.04 % for trypsin protease (Fig. - 16b).

**Fig. 12:**
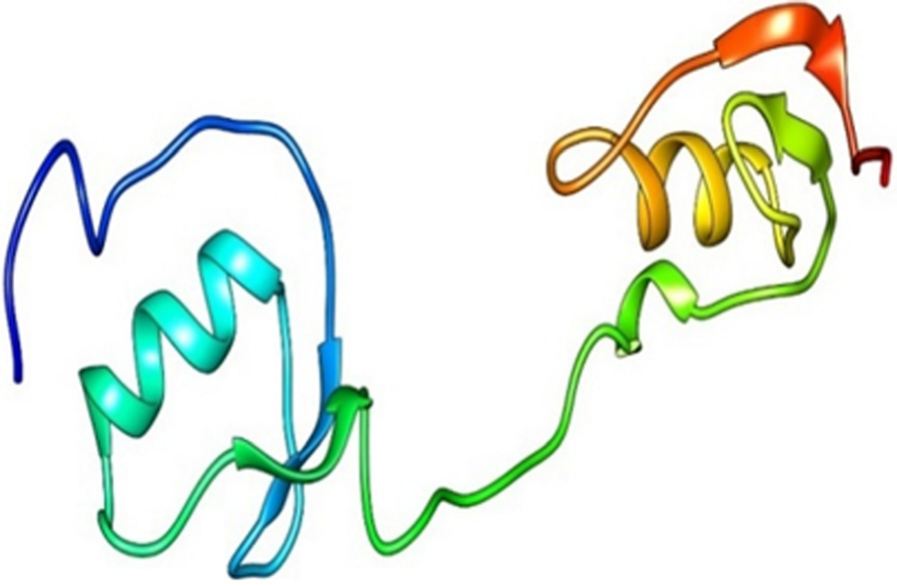
Modeled Structure of Kazal type Serine by Threading approach. Kazal type Serine protease was modeled using threading approach representing 4 helices and 3 sheets

**Fig. 13:**
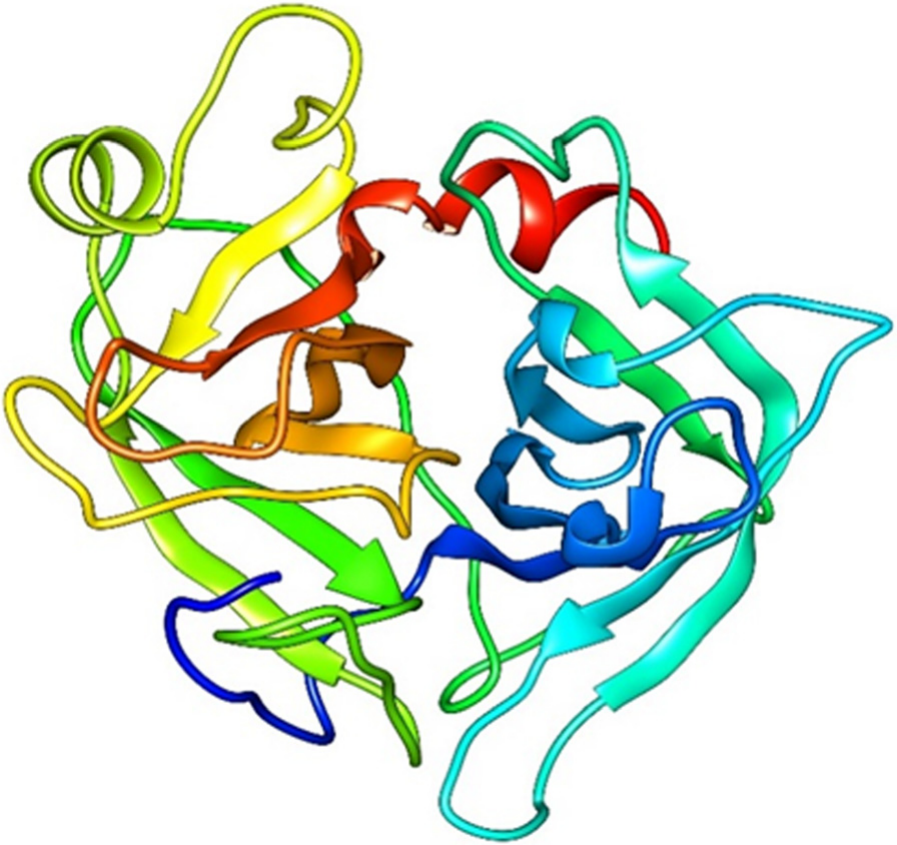
Modeled Structure of Trypsin protease by Threading approach. Trypsin protease modeled using threading approach represents 5 helices and 13 sheets

**Fig. 14:**
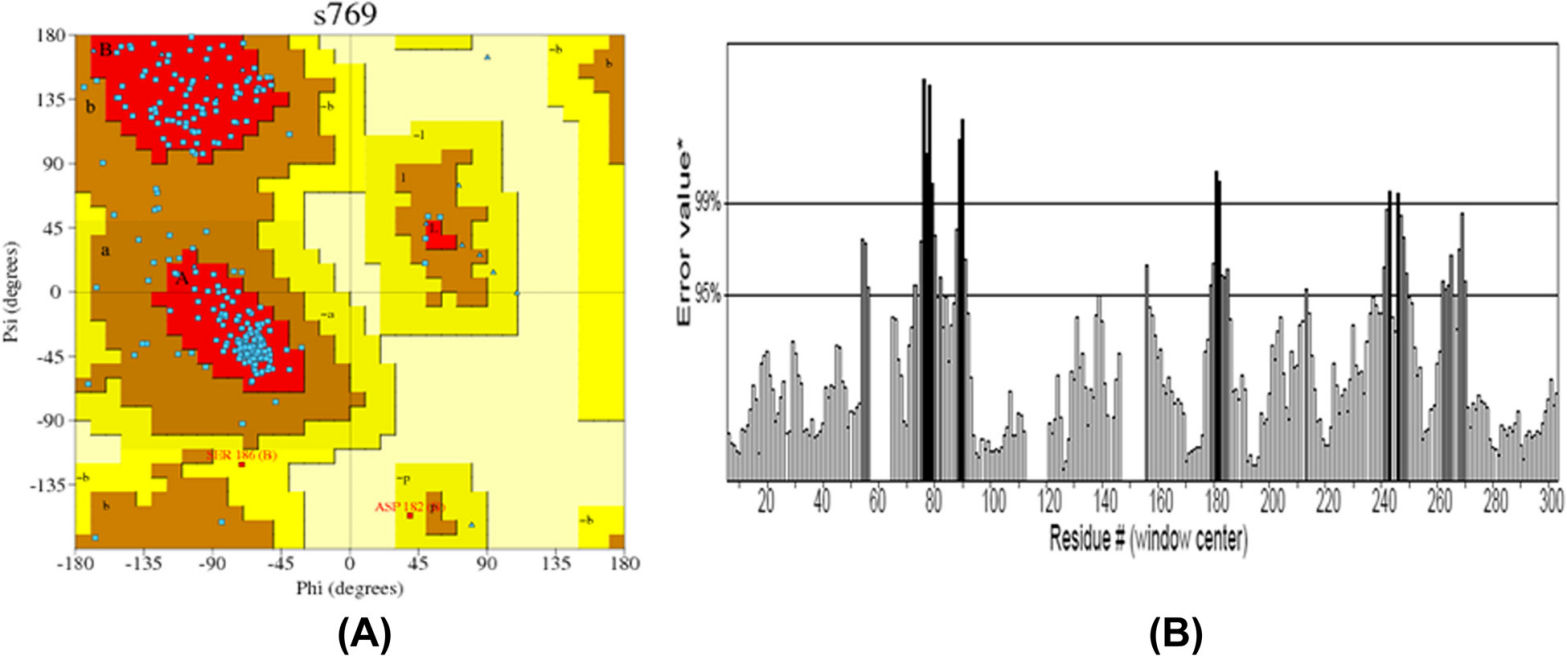
Model validation of SNTI by PROCHECK and ERRAT. (**a**) Ramachandran plot of SNTI represents 99.2 % of amino acids in favored region. (**b**) The overall quality of structure is 86.029 %

**Fig. 15:**
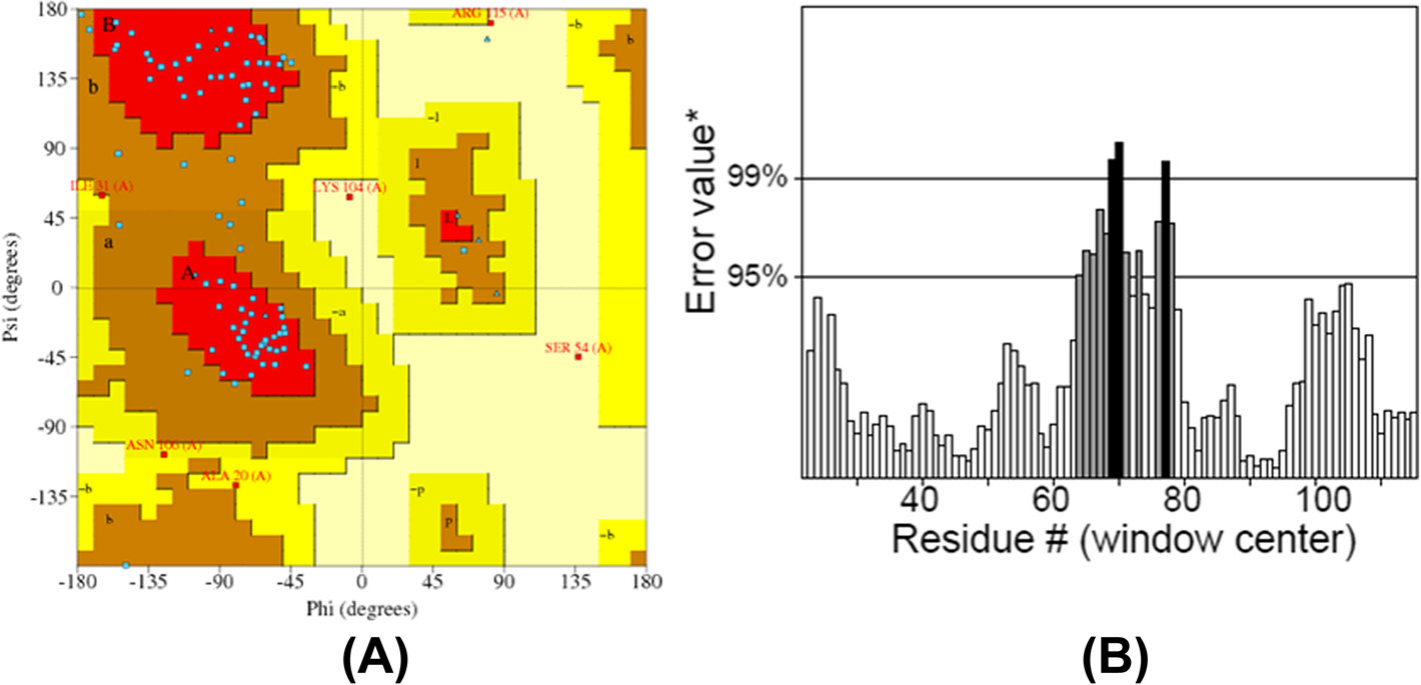
Model validation of Kazal type Serine protease by PROCHECK and ERRAT. (**a**) Ramachandran plot of Kazal type Serine protease represents 92.5 % of amino acids in favored regions. (**b**) The overall quality of structure is 86.96 %

**Fig. 16:**
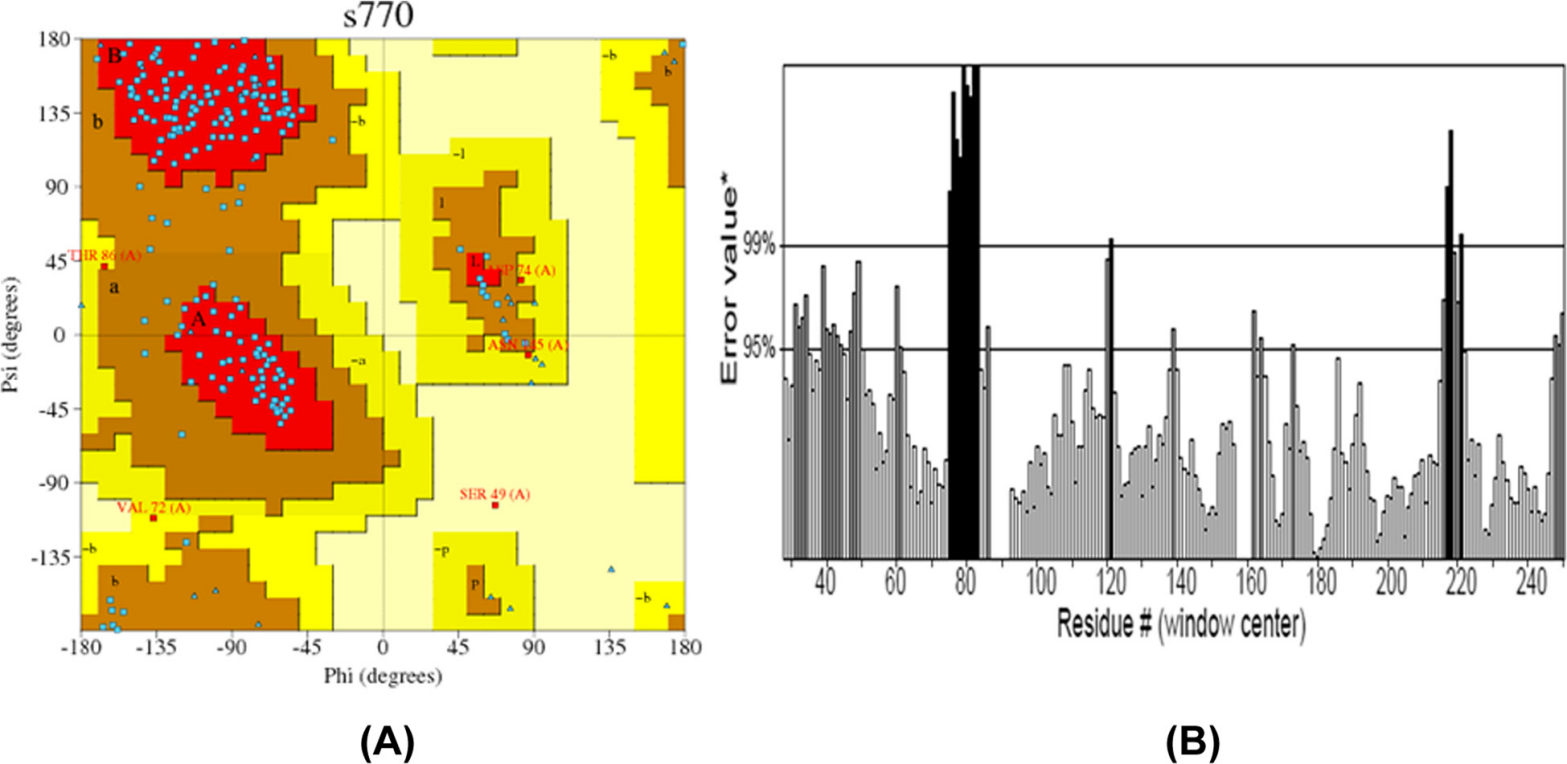
Model validation of Trypsin protease by PROCHECK and ERRAT. (**a**) Ramachandran plot of Trypsin protease represents 97.5 % of amino acids in favored regions. (**b**) The overall quality of structure is 81.04 %

 Binding Site prediction: The predicted structures were subjected to SiteMap for binding site identification. The hydrophobic binding sites predicted by SiteMap on the surface of SNTI and gut proteases are shown in the Table - 3.

Protein-Protein Docking: By following these combinations of SNTI x Kazal type Serine protease and SNTI x Trypsin protease was performed using PIPER. All these proteins prior to docking were prepared, optimized and energy minimized. From the resultant set of 10 poses the hydrogen binding interactions and other interactions were identified (Figs. – 17, 18). They are further checked whether these interactions are present in predicted binding sites. Table – 4 represents interacting residues of SNTI x Kazal type Serine protease complex and SNTI x Trypsin protease that are involved in binding site regions.

**Table 3:** Binding site surfaces predicted by SiteMap

Protein	Residues present in hydrophobic surface
SNTI	Asp 2, Lys 10, Val 14, Thr 79, Lys 299, Asn 90,
Kazal type Serine	Leu 70, Pro 71, Gly 75, Met 80
Trypsin	Gly 73, Ala 76,Arg 80, Gly 160,

Amino acids present in the hydrophobic binding surface of SNTI, Kazal type Serine and Trypsin

**Fig. 17:**
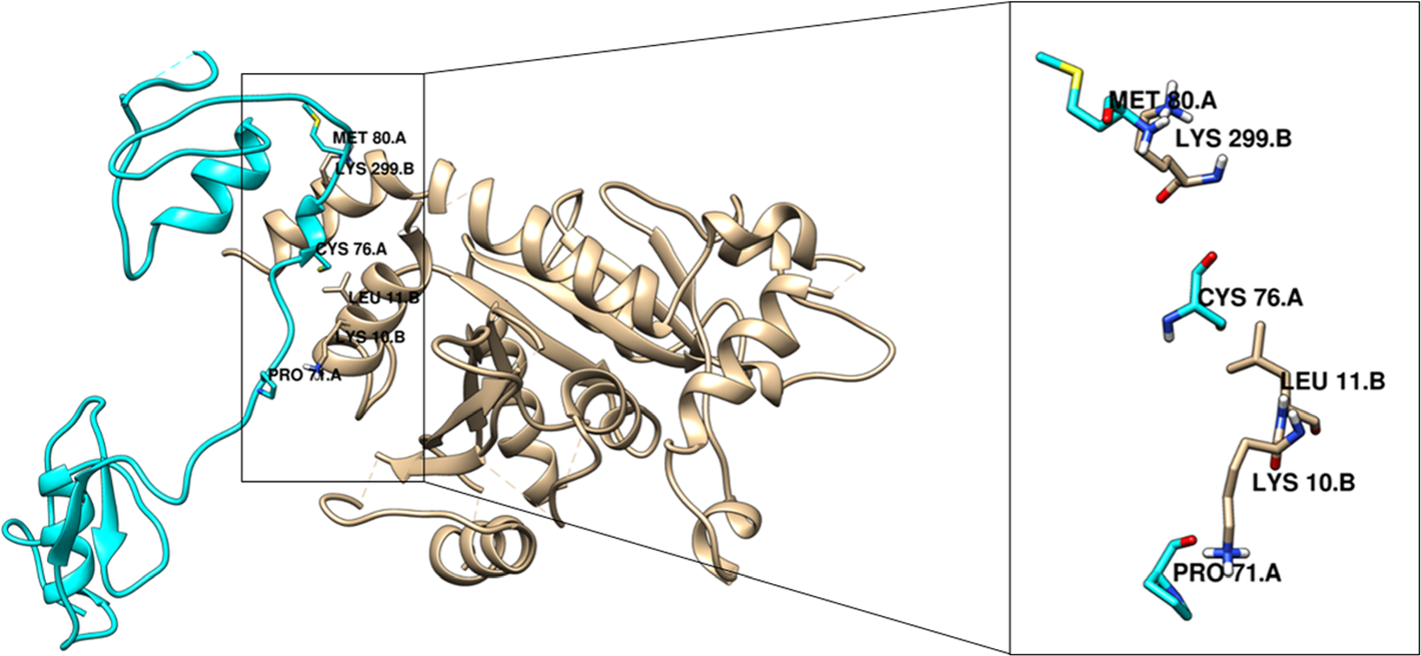
Protein-Protein interactions of SNTI with Kazal type Serine protease from *H. armigera.* Cyan color residues represents Kazal type Serine and Tan color residues represents SNTI

**Fig. 18:**
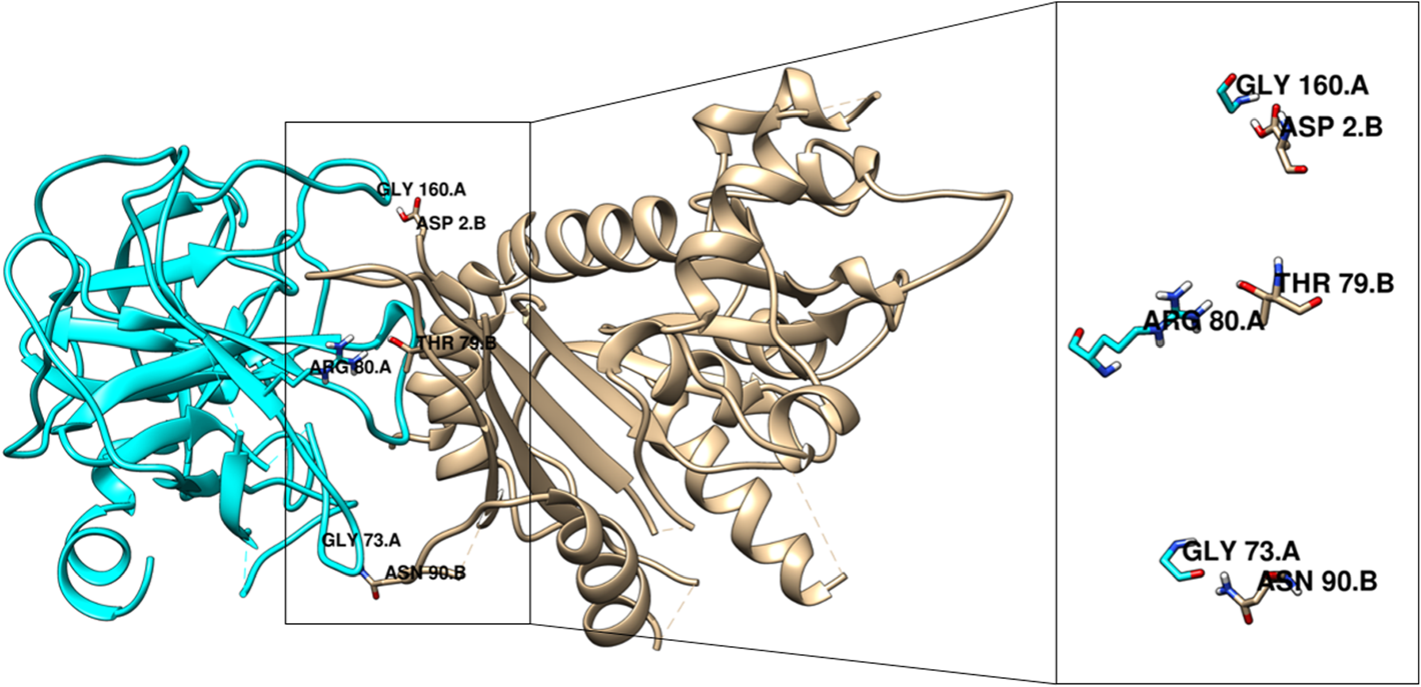
Protein-Protein interactions of Kazal type Trypsin. Cyan color residues represents Trypsin and Tan color residues represents SNTI

**Table 4:** Protein-protein interaction analysis of SNTI with Kazal type Serine protease and SNTI with Trypsin

Protein-protein docking	Residues interacted	Distance in Å	Type of interaction
SNTI - Kazal type Serine protease	Lys 10 – Pro 71	3.1	Hydrogen Binding
	Lys 299 – Met 80	3.1	Hydrogen Binding
	Lue 11 – Cys 76	3.8	Van Der Waals
SNTI – Trypsin protease	Thr 79 – Arg 80	3.1	Hydrogen Binding
	Asn 90 – Gly 73	3.0	Hydrogen Binding
	Asp 2 – Gly 160	2.9	Hydrogen Binding

## Discussion

The observation that the trypsin inhibitory activity in the crude extracts of these seeds is stable at 700C for 10 min has led to the use of this treatment as the first step in the purification of the inhibitor. This step also helps in the removal of the endogenous proteolytic activity present in the seed extracts [32]. About 50 % of the proteins present in the crude extract were removed by this step. When the ammonium sulphate fraction was subjected to CM-cellulose column chromatography, protease inhibitory activity was found to be associated with the protein eluted from the column by 0.3M NaCl. SNTI has been found to be homogeneous by the criteria of native PAGE and gel filtration. HPLC analysis revealed a single peak which represents the purity of the compound.

Protease inhibitors have been separated by several gel electrophoretic methods. Anionic inhibitors have been examined by the Davis method [43], cationic inhibitors by the Reisfeld method [32] and neutral inhibitors by Weber and Osborn [44] and Laemmli [45] methods. While these methods have proved useful for establishing the purity and determining the molecular weight of proteins, they cannot be used to distinguish isoinhibitors or compare the activity of the particular inhibitor against different proteolytic enzymes. SNTI in all the fractions during its purification showed a single inhibitory band specific to trypsin on gelatin PAGE. A single inhibitory band also signifies the absence of isoforms which are common in many sources [46, 47, 48].

The molecular weight of SNTI as determined by SDS-PAGE was 29 kDa.  This is close to the value 28.5 kDa obtained for the inhibitor by gel filtration on Sephadex G-100. Some protease inhibitors have exhibited anomalous behavior on Sephadex gel columns due to the existence of oligomeric forms of inhibitors arising from monomer– dimer – trimer equilibrium [49, 50, 51] or due to the presence of carbohydrate moieties. The subunit nature of SNTI has been analyzed by the SDS – PAGE technique. The inhibitor showed a single sharp band on SDS – PAGE when stained with silver supporting the monomeric nature of the protein. Further SNTI did not give positive result with PAS stain suggesting it free from carbohydrate moieties. Most of the trypsin inhibitors are non-glycoproteins. Papain inhibitor from potato tubers is a glycoprotein with a molecular weight of 80 kDa [52]. Shakuntala [53] first identified a glycoprotein trypsin inhibitor from Jack fruit seeds and to possess lectin activity.

The unusual stability of protease inhibitors, in general, is their most remarkable property. SNTI showed similarities to other protease inhibitors from soy bean [54] in their stability. The low cysteine content [35] in these inhibitors negates the possibility of the stability of the inhibitors rendered due to extensive intra-peptide cross - linking. However, the unusual stability of the inhibitor may be due to strong hydrophobic interactions forming an inner core in the protein.

The result of the investigation of the inhibitory specificity of SNTI has shown it to be a serpin and is strongly active against bovine trypsin and porcine elastase. SNTI was ineffective against other proteases such as papain (thiol), pepsin (carboxyl) and thermolysin (metallo). Majority of plant protease inhibitors isolated so far have been found to be specific for serine proteases [55].  However, there are reports of plant protease inhibitors inhibiting other classes of proteases. The trypsin/ chymotrypsin inhibitor from broad beans inhibits the sulphydryl enzyme papain [56].  Serine protease inhibitors such as barley subtilisin inhibitor [57] and wheat germ protease K inhibitor [58] are found to be active against α – amylases. The human LEKTI has 15 domains and inhibits plasmin, trypsin, elastase, subtilisin A and cathepsin G [59].

As regards the mechanism of action, SNTI has shown a non-competitive type of inhibition. Although a few like soybean trypsin inhibitor has shown the competitive type of inhibition, the majority of the inhibitors follows non-competitive inhibition kinetics [46]. Jack fruit seed protease inhibitor isolated by Annapurna et al., [42] also showed non-competitive enzyme inhibition but the one isolated by Bhat [60] exhibited uncompetitive inhibition. The Ki value of SNTI was found to be 0.75+0.05x10-10 M.  The low Ki value indicates high affinity of SNTI towards trypsin.

The formation of stable trypsin inhibitor complex has been demonstrated by Sephadex G-100 gel filtration studies. The results obtained suggest that the inhibitor binds to trypsin in a 1:1 molar ratio. SNTI is a mono-headed inhibitor with a site for trypsin. Double-headed inhibitors with overlapping or non-overlapping binding sites are reported from plant sources [42, 53].

Protein modelling is now widely used in docking studies which paved way for the availability of disease causing target proteins in living organisms. SNTI has a stretch of 278 amino acids and the gut proteases from H. armigera and S. frugiperda are 119 and 254 amino acid residues respectively. In the present study two different modeling approaches homology and threading were used based on the availability of the template. The protein SNTI was modeled using the homology modeling approach as the sequence similarity between the template and target is more than 40 % whereas for the gut proteases from insects the structure was modeled using threading approach. The main difference between the homology modeling and threading is that in homology modeling the structure is built based on the sequence of the template whereas in threading it uses the knowledge of folds. Upon validation of the modeled proteins the structures are found to be reliable for subjecting to docking as about more than 80 % of amino acids fall in favorable regions of Ramachandran plot. The protein-protein docking and interaction analysis have indicated that the interacting residues between the surfaces of the docked proteins are the same residues that were predicted by Site Map. As the interactions involves the binding site residues with strong hydrogen bond and Van der Walls forces, these studies indicate that SNTI have shown potent activity against the gut proteases. Further evaluation of the study using wet lab techniques could bring out a natural source of bio-pesticide.SNTI belongs to the Serine Cereal super family and was found to exhibit both anti-bacterial and anti-fungal activity [61]. SNTI was reported to have anti-bacterial and anti-fungal activity but till date no adequate literature is available on insecticidal activity of SNTI, but SSTI was shown to exhibit insecticidal activity. As SNTI was reported to have anti-bacterial and anti-fungal activity, it might also possess insecticidal activity and hence was evaluated using various in silico tools. The sequence of SNTI obtained from MALDI-TOF [35] and the gut proteases of H. armigera and S. frugiperda were subjected to modeling. The resultant structures were validated and then subjected for protein-protein docking to understand the inhibitory role of SNTI on larval gut proteases.

## Conclusion

Results demonstrate that SNTI is a very stable, purified and highly potent trypsin inhibitor. Inhibitors of proteinases have been successfully engineered for protection of plants against pests and microorganisms. Protease inhibitors are proficient in interfering with digestive enzymes of insect gut and hence are able to control them. The overall structural quality of three proteins SNTI, Kazal type serine protease and trypsin protease validated by ERRAT server was found to be 86.02, 86.96 and 81.04 %. Docking results reveal that SNTI strongly interacts by hydrogen bonds and Vander Waals forces with the gut proteases at their active sites. SNTI binds at the active sites of gut protease enzymes which renders them inactive. This blocks the process of breaking down of nutrient proteins thereby causing malnourishment of the larvae leading to lethality. Hence Protease inhibitors can be commonly used as natural bio-pesticides in controlling pests.

Further analysis of structure, protein-protein interactions and diverse biological activities of SNTI on different proteases of diverse biological origins need to be carried out to confirm the biotechnological potential of SNTI as a bio control agent and its therapeutic potentials. Compromises between increased complexity, pharmacokinetic profiles, and drug affordability will challenge biochemists to find new general methods for the simple creation of new inhibitors, which are potent, selective and bioavailable or to find better methods for efficient delivery of protein inhibitors against proteases. We hope that this endeavor can help to stimulate new efforts towards achieving such goals.

## Abbreviations

FTIR: Fourier transform infra-red spectroscopy
SNTI: Soap Nut Trypsin Inhibitor
PI: Protease inhibitor
SDS-PAGE: Sodium dodecyl sulphate – polyacrylamide gel electrophoresis
PAS: Periodic acid schiff’s
PB: Phosphate buffer
